# Seismic Performance of Precast Drift-Hardening Concrete Walls Connected by Grout–Sheath Duct

**DOI:** 10.3390/ma17215165

**Published:** 2024-10-23

**Authors:** Jiayu Che, Shiyu Yuan, Yuping Sun

**Affiliations:** Graduate School of Engineering, Kobe University, 1-1 Rokkodai-cho, Nada, Kobe 657-8501, Japan; chejiayu003@163.com (J.C.); yuanshiyu@person.kobe-u.ac.jp (S.Y.)

**Keywords:** drift-hardening concrete wall, SBPDN rebar, precast, sheath duct, bond strength

## Abstract

In order to find a suitable size of sheath duct and a reliable construction method for precast walls, a cast-in-place and five 1/2 scale precast drift-hardening concrete walls reinforced with weakly bonded ultra-high strength SBPDN rebars were fabricated and tested under reserved lateral load and constant compression. The experimental variables were the diameter of sheath ducts (45 mm, 100 mm, and 120 mm), embedded length (20d and 35d; d is the nominal diameter of SBPDN rebars), axial load ratio (0.075 and 0.15), and the construction method. The experimental observations, hysteresis behaviors, envelope curves, residual deformation, crack propagation, and energy dissipation were compared in the study. Moreover, a formula was applied to calculate the bond strength of the sheath duct. The experimental and calculated results revealed that increasing the axial load ratio and embedment length could not enhance the bond strength of the sheath ducts, and increasing the diameter decreased the bond strength significantly. Anchored the SBPDN rebars in smaller sheath ducts separately was a more stable connection method for precast concrete shear walls and provided sufficient drift-hardening capability, even at a large drift level (over 3%).

## 1. Introduction

Reinforced concrete (RC) buildings are one of the most common construction types located in areas with frequent seismic activity, especially in Japan [1]. In Japan, the history of seismic design codes and laws for buildings can be traced back to 1924, when the Urban Building Law was revised as a consequence of the disaster of the great Kanto earthquake in 1923 [2]. The initial method of seismic design aimed to ensure the safety and serviceability of buildings during moderate earthquakes. Based on the lessons learned from the earthquakes during the 1960s and 1970s in Japan, in 1981, the Japanese building code underwent its most significant revision. A new second-phase seismic design methodology, which was generally referred to as the new seismic design method (NSDM) in Japan, was proposed to provide structures with sufficient ductility to avoid severe losses to life and property [3].

However, observations of earthquake damage around the world have revealed that while most ductile concrete structures can endure strong earthquakes without collapsing, the significant residual deformations make repairs nearly impossible. As a result, these damaged buildings and structures have to be demolished, leading to substantial human casualties and financial costs. Considering these reasons mentioned above, there is an urgent demand for engineers to control the damage caused by strong earthquakes and increase the repairability and restorability of structures and buildings.

Following the revision of the Architectural Institute of Japan (AIJ) code in 2010 [4], the proposed cross-sectional shape for concrete shear walls was officially recognized, and the allowable stress-design method for concrete shear walls without columns restrained was established. To overcome the drawbacks of ductile concrete structures mentioned above, Sun et al. [5,6,7] proposed a drift-hardening concrete (DHC) structure that can significantly enhance the seismic performance of building structures. Compared to ductile concrete structures, DHC structures exhibit two key characteristics: (1) The lateral resistance steadily increases with deformation, even at large drift levels (over 2%), indicating positive stiffness under large deformation. (2) Residual deformations are greatly suppressed with a clear origin-directed tendency, indicating good reparability. Because of these features, DHC structures maintain strong restorability and deformation capacities when subjected to unexpected major earthquakes. The application of DHC structures in buildings can potentially raise the current design standard from “minor tremors without damage, moderate tremors with reparability, and major tremors without collapse” up to “moderate tremors without damage, major tremors with reparability”.

On the other hand, similar to cast-in-place (CIP) walls, precast walls (PW) are also highly regarded in the engineering community due to their construction efficiency and quality, energy efficiency, and emission reduction [8,9]. Therefore, the use of precast components in practical applications has steadily increased and gained widespread adoption in recent years [10,11,12]. A strong and reliable connection between the wall panel and foundation plays an important role in determining the seismic performance of PWs. An unstable connection method may significantly reduce the overall deformation capacity and reparability of the building structures, potentially leading to their demolition and resulting in substantial economic waste.

Currently, there are numerous structural forms of research concerning reinforced concrete (RC) shear walls. Based on whether secondary concrete casting is required, they can be divided into two main categories: dry connection and wet connection [13]. The dry connection is typically assembled from several precast concrete components using reliable fastening methods, such as prestressed rebars or bolts, to form a unified component. On the other hand, the wet connection generally employs rebar or steel plate anchoring overlaps or mechanical connections combined with concrete or mortar to form an integral joint.

For the dry connection, Kurama [14] proposed an unbonded post-tensioned (UPT) precast concrete shear wall and introduced a performance-based seismic design (PBSD) method based on its deformation and damage [15]. Based on the PBSD, many scholars have conducted extensive in-depth analysis and research on various connection systems utilizing UPT construction, such as open-hole shear walls [16], limb structures [17,18], and vertical seam connections [19], forming a relatively complete design method for a UPT precast shear wall system. Bora [9] used a long-threaded rod connection technique to connect the PW panel to the foundation beam. Due to the joint of PW relying on mechanical anchoring for stability, the sliding effect cannot be ignored, making the overall deformation of structures relatively large. At present, the dry connection is mainly used in multi-layer shear wall structures, but the applicability for taller buildings still needs further research.

Grout–sheath duct connections, as a representative method of wet connection, are now widely used around the world. In the 1990s, Japanese scholars conducted comprehensive experimental research on this connection method and proposed a load-carrying capacity calculation formula based on the constant bond strength assumption in the connection [20,21,22]. In recent years, numerous scholars worldwide have investigated the performance of grout–sheath duct connections under various conditions like different anchorage lengths, diameters of sheath ducts, grouting construction methods, and materials [23,24,25,26,27]. Yang [28] examined the coefficients to ensure the safety of the grouted joint. Nicoletti [29] proposed a graphical tool to estimate the proper size of the joint in the early design stage. Tang [30] proposed a novel connection with a spiral stirrup-restrained grout-anchored lapped reinforcement. Wang [31] applied the grout–sheath duct connection with central-tenon enhancement for precast piers.

Since the splicing areas of PWs are much larger than those of precast beams and columns, when the PWs are subjected to lateral loads, through-cracks can easily occur at the splice area, resulting in the damage of connections, thereby affecting the overall structural seismic performance. Wu [32] conducted experimental and theoretical research on RC short-leg shear walls with grout–sheath duct connection, and the results show that the bearing capacity of PW is essentially similar to that of CIP shear walls, but the ductility and energy dissipation are slightly lower.

From the perspective of improving the practicality of the DHC walls and based on the research findings mentioned above, this study intends to develop precast DHC walls and establish a reliable connection method between the wall panel and foundation beam using a grout–sheath duct. The objectives of this paper are as follows: (1) To investigate the influence of the size of the sheath duct and construction method on the seismic performance of the DHC walls in different axial load ratios (ALRs). (2) To verify the drift-hardening capability of the precast DHC walls. (3) To confirm the bond strength of sheath ducts with different sizes. (4) To compare the energy-dissipation performance of PWs and CIP walls connected by different sizes of sheath ducts.

## 2. Experimental Program

### 2.1. Specimen Preparation

In order to achieve the objectives mentioned above, a total of six 1/2 scale DHC walls were fabricated and tested using a reversed lateral load under constant axial load. Among the six DHC walls, a CIP wall was set as the control item, while the other five PWs were designed with different sheath duct diameters (45 mm, 100 mm, and 120 mm), embedded lengths (20d and 35d; d is the diameter of concentrated SBPDN rebars), construction methods, and axial load ratios (0.075 and 0.15).

**Figure 1:**
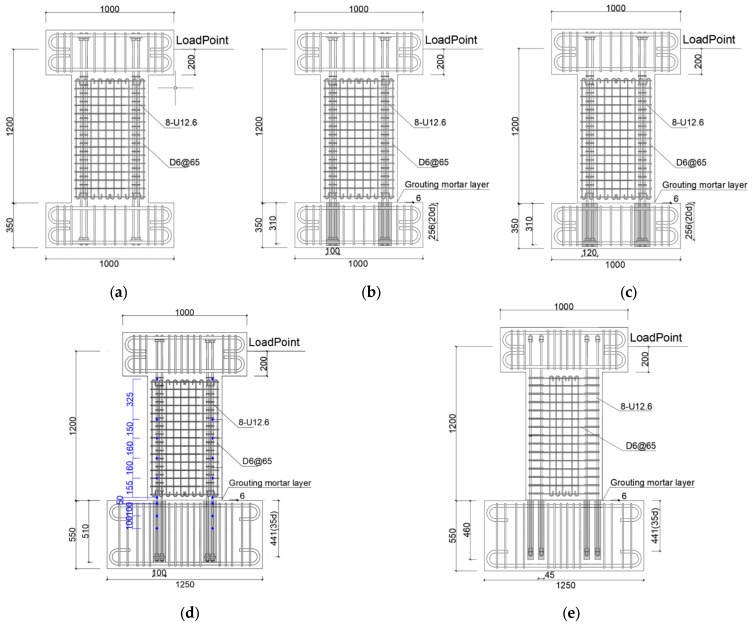
Dimensions and reinforcement details: (**a**) W20d; (**b**) WP20d-100 and WPH20d-100; (**c**) WP20d-120; (**d**) WP35d-100; (**e**) WP35d-45.

**Figure 2:**
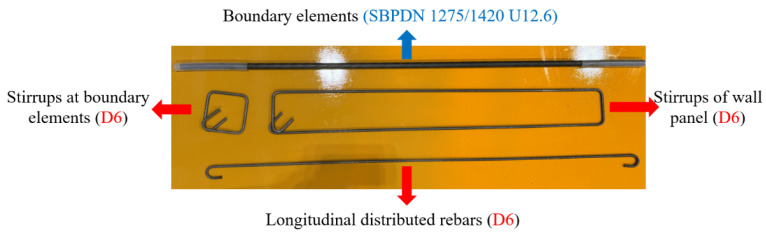
Reinforcement used in the DHC walls.

As can be seen in Figure 1 and Table 1, the concentrated rebars consisted of four weakly bonded ultra-high strength SBPDN 1275/1420 U12.6 rebars (yield strength and ultimate strength are 1275 MPa and 1420 MPa, respectively, hereafter referred to as SBPDN rebars) and fixed as the boundary elements of DHC walls. Both the longitudinal and transverse distributed bars of the wall panel consisted of SD295A D6 (hereafter referred to as D6 rebars) deformed rebars with a spacing of 65 mm. Since specimens were not fabricated at the same time, the used rebars were in different batches, which were distinguished by “-1, -2 and -3” in Table 1. Moreover, to prevent concrete from being damaged in the early stage and control the construction difficulty of grouting operation, the longitudinally distributed rebars were not extended into the foundation beams, and the reinforcement used in the wall panel is summarized in Figure 2. The #1000-series galvanized steel sheets sheath ducts, which comply with the Japanese standard JIS G3302, were applied as connection between wall panels and foundation. All the DHC walls had a rectangular section of 600 mm in length and 150 mm in thickness, 1200 mm from foundation beam to loading point to give specimens the shear span ratio of 2.0.

In the names of specimens (e.g., WPH20d-100), the combination of letters and numbers for WPH20d indicates precast shear wall, with the embedded length of rebar being 20 times diameter of SBPDN rebars and loaded with a higher ALR (the ALRs of other five specimens are all 0.075, so it has been omitted). Further, -100 indicates that the diameter of the sheath duct is 100 mm.

### 2.2. Grouting Operation and Material Properties

**Figure 3:**
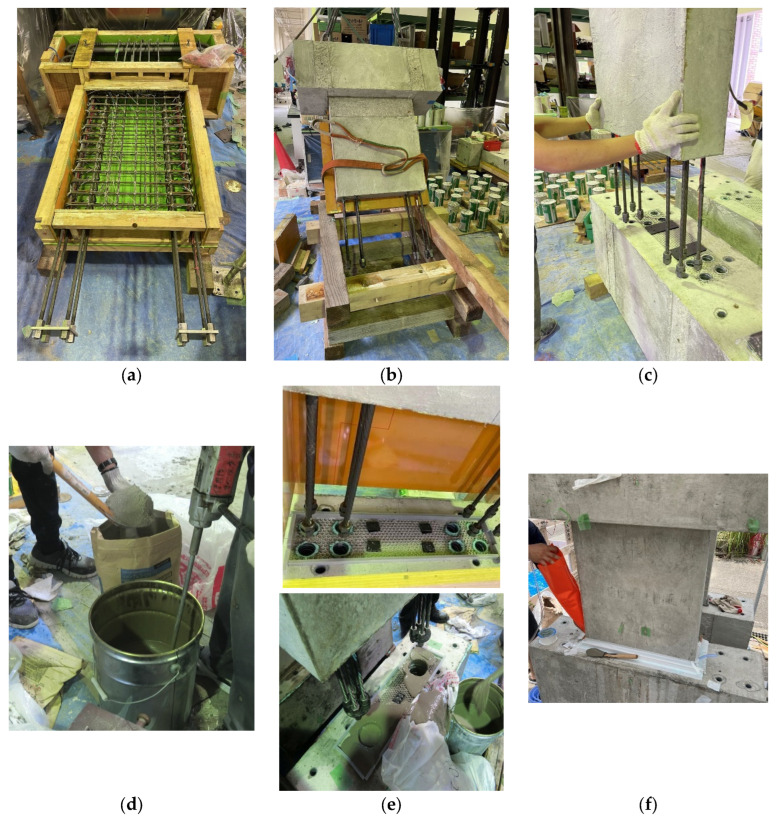
Construction details of fabricating DHC walls: (**a**) formworks assembly; (**b**) wall panel lifted by crane; (**c**) adjustment of the position of combining; (**d**) grout mixing; (**e**) injection of grout; (**f**) injection of grout to the interface after combining.

Figure 3 represents the process of the grouting operation. As can be seen in Figure 3a, the wall panel was cast as the upper part of the specimen together with the upper foundation beam. Seven days after concrete placement, the upper part of the specimen was lifted by a crane, and SBPDN rebars were anchored into the sheath ducts in the lower foundation beam (see Figure 3b,c). A cementitious non-shrinkage mortar that excels in fluidity, named PREU-LOX, was used as the grouting material for this test and mixed using a hand mixer for over 120 s, as shown in Figure 3d. According to Figure 3e, to prevent grout overflow, when the injected grout reached 80% of the volume of sheath duct, the crane was slowly controlled to bring the wall panel into full contact with the foundation beam, and squeeze bag (orange bag shown in Figure 3f) was used to inject the grout to the remaining part of boundary surface.

The boundary surface between the wall panel and the foundation beam was roughened with bubble wrap before casting (see Figure 3e). Moreover, to ensure the flowability and compactness of grout, the wall panel was raised 6 mm above the surface of foundation beam, and an air vent was set at the farthest point of the boundary surface where the grout was injected (see Figure 3f).

**Figure 4:**
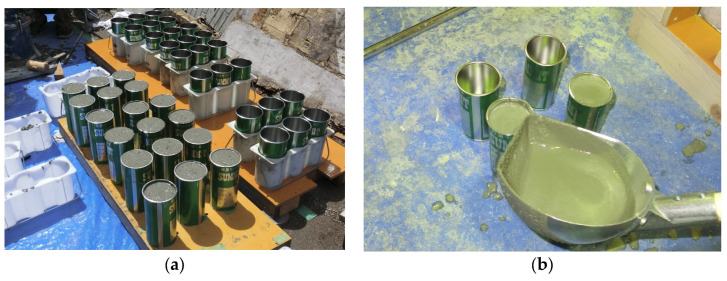
Cylinders of concrete and grout: (**a**) concrete (ϕ100 mm); (**b**) grout (ϕ50 mm).

To confirm the compressive strength of concrete and grout, three *ϕ*100 mm concrete cylinders and three *ϕ*50 mm grout cylinders of each specimen were cured under the same condition, as shown in Figure 4. The average value of the measured compressive strength for each specimen is listed in Table 1.

**Figure 5:**
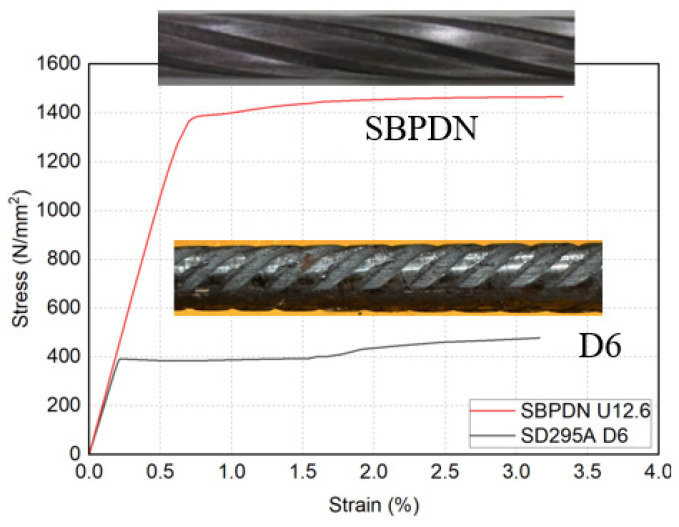
Stress–strain relationships of the reinforcements.

As the boundary elements of DHC walls, SBPDN rebar has spiraled grooves on its surface, as shown in Figure 5. According to previous research by Funato [33], when embedded in concrete with compressive strength of about 40 MPa, the bond strength of U12.6 SBPDN rebar was about 3.0 N/mm^2^, which is about one-fifth of that of deformed rebar. The tensile stress–strain curves, together with mechanical properties of the used rebars, are summarized in Figure 5 and Table 2.

### 2.3. Loading Protocol

**Figure 6:**
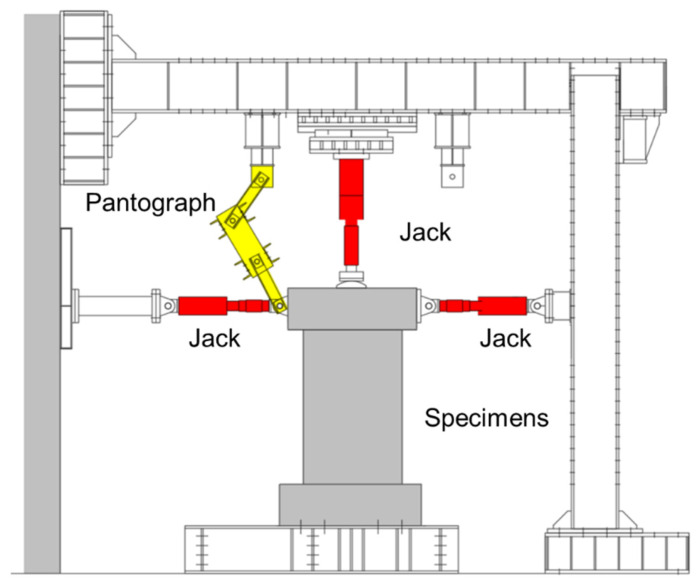
Schematic of experimental setup.

**Figure 7:**
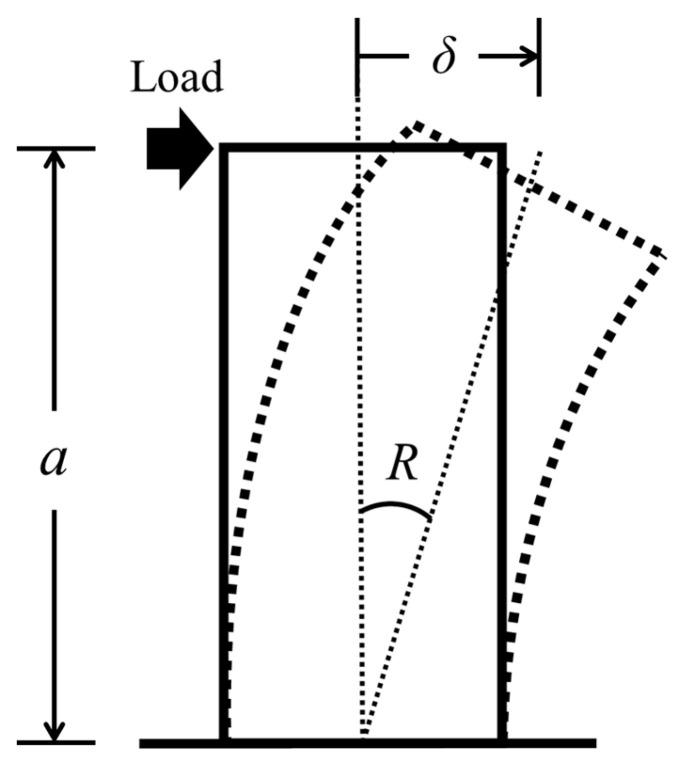
Definition of drift ratio R.

**Figure 8:**
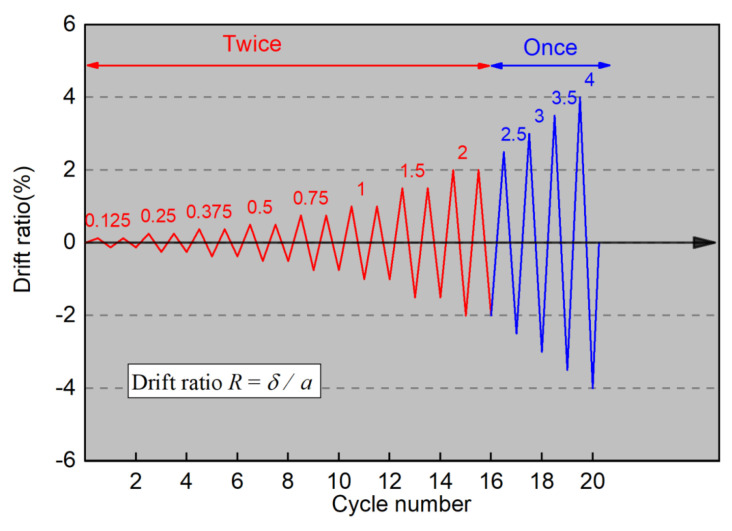
Loading program.

Figure 6 shows the schematic of experimental setup. The axial compression was applied by a 1000 kN hydraulic jack, and the cyclic lateral loading was controlled by two 300 kN jacks. The horizontal jacks were connected to reaction frame by hinge joints, and the cyclic lateral loading was controlled by the drift ratio (R), which is the ratio of the lateral displacement (*δ*) to the shear span (*a*) of the wall (see Figure 7). The loading program, which has been widely applied in Japan, is displayed in Figure 8. As shown in Figure 8, the load cycles were applied for two completely reversed cycles for each targeted drift ratio within 2% (including 2%) and one complete cycle for the drift ratio beyond 2%. The test was terminated when the walls could no longer sustain their own gravity or when the lateral resistance was reduced to 85% of the peak load.

### 2.4. Instrumentation and Measurement

**Figure 9:**
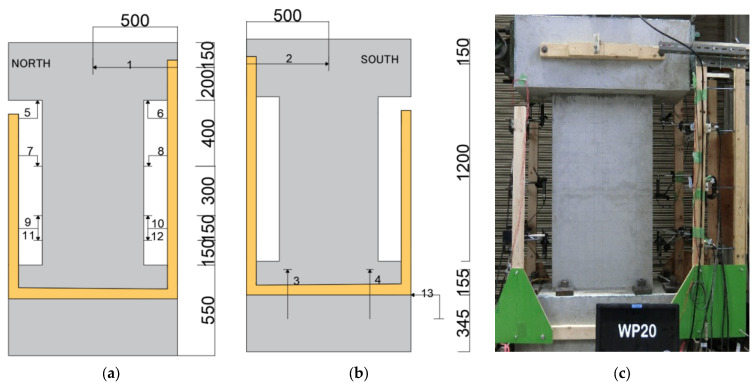
Locations of displacement transducers (DTs): (**a**) front view of the DTs; (**b**) back view of the DTs; (**c**) overall view of a testing specimen.

The displacement transducers (DTs) and the overall view of a test specimen are displayed in Figure 9. Lateral displacement is defined as the relative lateral displacement of the loading point with respect to the stub at the wall toe and was measured by DTs 1 and 2, which were set at the loading point through fixtures attached to the foundation beam. DTs 5 and 6 were set to measure the overall axial deformation of the wall panel. The axial deformation of the wall assumed that the foundation beam was totally rigid. DTs 7 to 12 were set to measure the axial deformation within the range of the wall toe to the heights of 150 mm, 300 mm, and 600 mm. DTs 3 and 4 were attached to check the rotation of the foundation, and DT 13 was attached to confirm the lateral displacement of the foundation.

The strain gauges were attached to the SBPDN rebars, as shown in Figure 1d, on both tensile and compressive flange sides of the DHC walls, and the locations were as follows: −225 mm, −125 mm, −25 mm, 25 mm, 180 mm, 340 mm, 500 mm, 650 mm, and 975 mm (boundary between wall panel and foundation was set as the critical point for the positive and negative limits).

## 3. Experimental Observation

**Figure 10:**
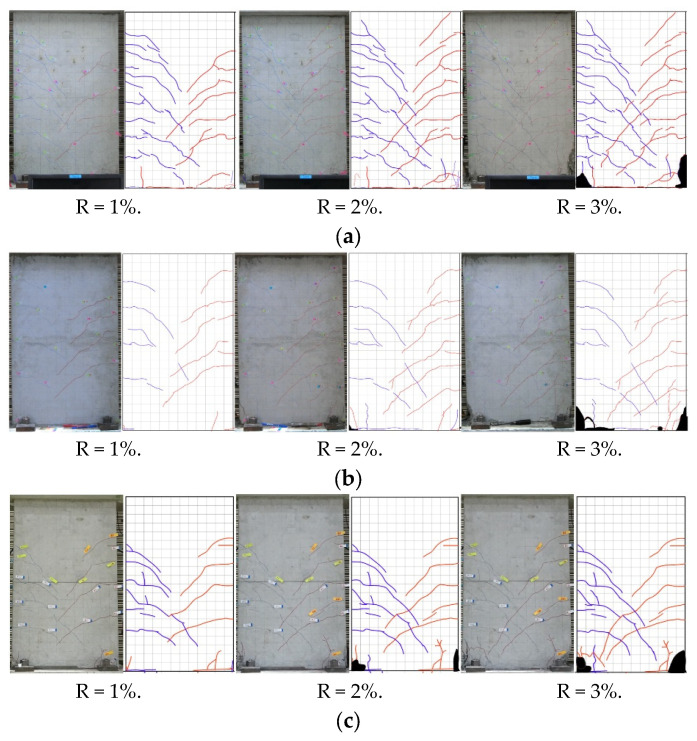

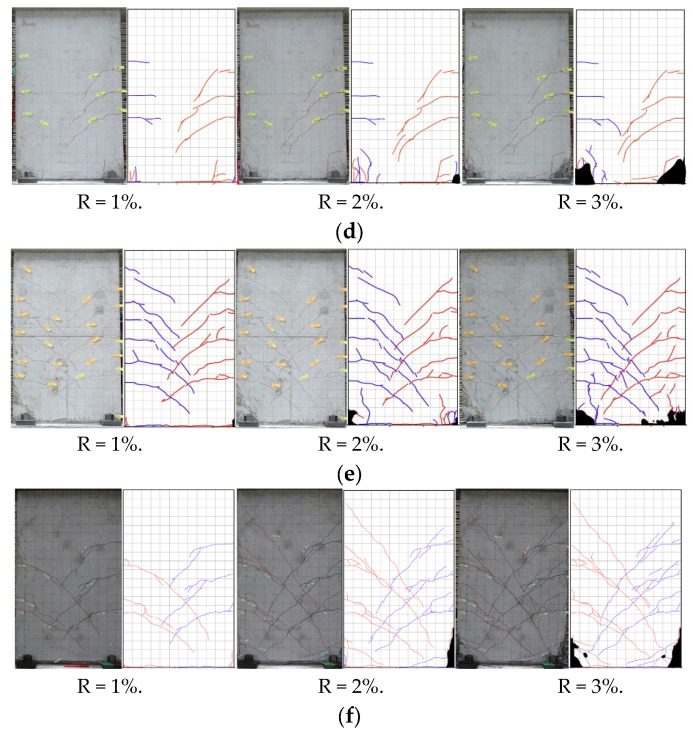
Propagation of cracks observed: (**a**) W20d; (**b**) WP20d-100; (**c**) WPH20d-100; (**d**) WP20d-120; (**e**) WP35d-100; (**f**) WP35d-45.

Figure 10 indicates the crack propagation of specimens discussed in this study. According to Figure 10, the position of the crack could be located by grids with sides of 50 mm. The red and blue lines represent cracks that occurred from the push and pull direction, respectively (the red and blue lines of specimen WP35d-45 have the opposite meaning to the other specimens). In addition, the black-filled portion was applied to indicate the concrete spalling off of the wall panel.

For specimen W20d, an initial flexural crack was confirmed at the wall toe when the drift ratio reached 0.125%. The crushing of concrete occurred at the compressed side of the wall toe with a drift ratio of 0.75%, and spalling was observed at 2%. As the drift increased, cracks progressively developed, leading to the shear failure of the specimen when the drift ratio approached 4%.

**Figure 11:**
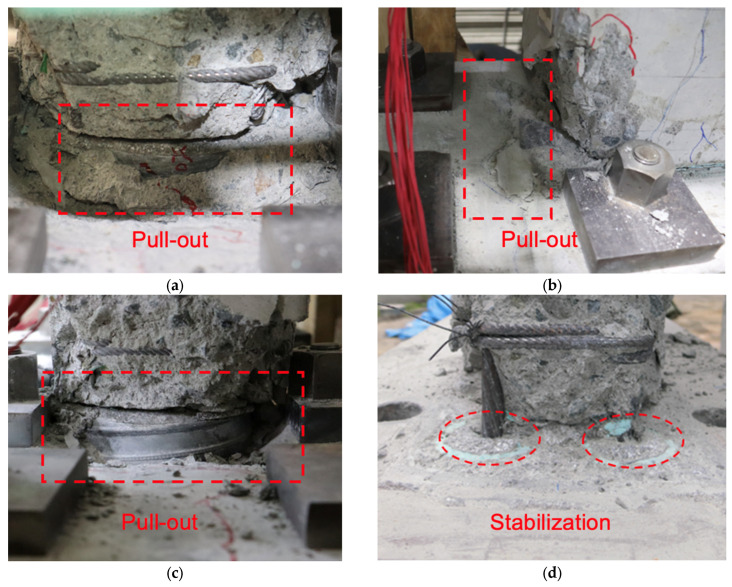
Observations of sheath ducts: (**a**) WP20d-100 (R = 3%); (**b**) WPH20d-100 (R = 2%); (**c**) WP20d-120 (R = 3%); (**d**) WP35d-45 (post-experiment).

For specimens WP20d-100, WPH20d-100, and WP20d-120, the flexural and shear cracks were confirmed with a drift ratio of 0.125%. As with W20d, damage to concrete also occurred at a drift ratio of 0.75%. When the drift ratio raised up to 1.5%, serval cracks were confirmed on the top surface of the foundation beam, which demonstrated that the sheath duct started to be pulled out from the original position. In addition, as can be seen in Figure 10a–d, the crack development rate of all three precast specimens was much slighter than that of CIP specimen W20d due to the damage being concentrated on the grout layer between the wall panel and foundation beam. The pullout became significant with the increasing deformation of specimens (see the areas enclosed by red dashed lines in Figure 11a–c).

For specimen WP35d-100, flexural and shear cracks were confirmed when the drift ratio approached 0.125%. The flaking of concrete emerged on the foundation beam, which indicated that the connection became unstable when the drift ratio raised to 2.5%. As the development of deformation, cracks, and concrete spalling occurred on the surface of foundation beam and the grout layer became significant, the test was terminated.

For specimen WP35d-45, flexural and shear cracks were confirmed when the drift ratio approached 0.125%. Concrete spalling-off emerged on the compression side since the drift ratio raised to 0.75%. Grout spalling occurred at the boundary layer between the wall panel and foundation, beginning with a drift ratio of 1%. As the deformation developed, the grout and concrete severely crumbled and then failed in shearing. Figure 11d reveals the post-experiment damage on the top surface of the foundation beam, and the dashed red lines indicate that sheath ducts were fixed at the original position. It is demonstrated that SBPDN rebars anchored in smaller sheath ducts separately could connect the wall panel to the foundation beam stably until the end of the load test.

## 4. Test Results and Discussion

### 4.1. Hysteresis Behavior

**Figure 12:**
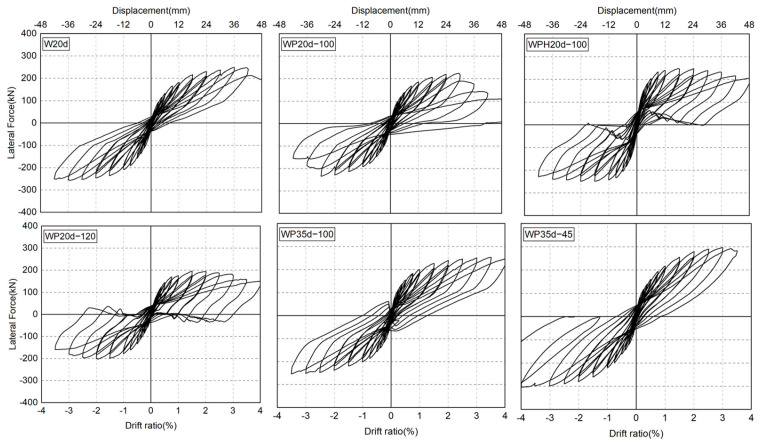
Measured lateral force versus drift ratio relationships.

Figure 12 represents the lateral force–drift ratio curves of all six specimens. The loading-carrying capacity of specimens W20d and WP35d-45 continued to increase with deformation until a drift ratio of 3%. The lateral resistance can still maintain 85% of the peak load at a drift ratio of 3.5%, demonstrating good deformation capability. It is clear that anchoring SBPDN rebars in sheath ducts separately is a stable approach for connecting a wall panel and foundation beam.

**Figure 13:**
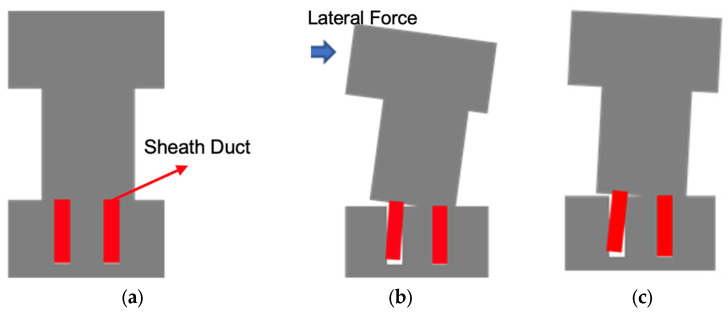
Schematic diagram of sheath duct being pulled out: (**a**) original state of sheath duct; (**b**) state of sheath ducts during loading; (**c**) state of sheath ducts after unloading.

For specimens that anchored SBPDN rebars in large sheath ducts (WP20d-100, WPH20d-100, WP20d-120, and WP35d-100), the lateral force barely increased since the sheath ducts were pulled out in a drift ratio of 1.5% (WP35d-100 in 3%). At the same time, during the stage of unloading, the curve reached 0 kN and then rebounded in the opposite direction. It could be implied that the sheath duct was pulled out during the loading stage of the test. However, owing to the ribs getting stuck in the concrete, the sheath duct could not return to the origin position when the specimen was unloading (sheath ducts are represented by red rectangles as shown in Figure 13). When the specimen was loaded in the reverse direction, the sheath duct started to move and released the force suddenly.

### 4.2. Envelope Curves

#### 4.2.1. Hysteresis Curves

**Figure 14:**
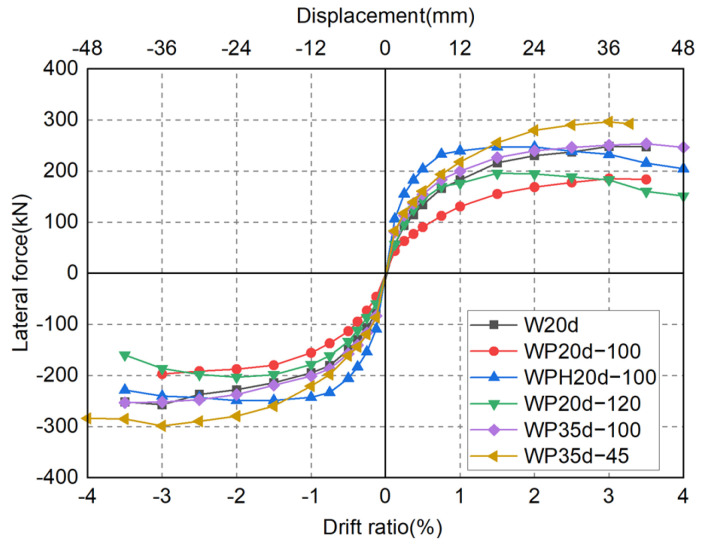
Envelope curves of hysteresis loops.

In order to clearly see the effects of experimental variables on the overall seismic behavior of precast DHC walls, the main experimental parameters of specimens and the envelope curves of hysteresis curves are summarized in Table 3 and Figure 14, respectively.

As can be seen in Table 3 and Figure 14, due to the high axial load ratio, the initial stiffness of specimen WPH20d-100 was much greater than that of the five other DHC walls. This was because a higher axial load ratio enhances the interlocking effect of aggregates within the wall, suppressing the occurrence and development of flexural and shear cracks, thereby increasing the initial stiffness and bearing capacity of the walls. Specimen WP35d-100 could trace the CIP specimen until a drift ratio of 3.5% with very good accuracy. However, repairability was reduced because the connection became unstable under large deformation. Because the connection became severely damaged in the early stage of the loading test, the loading-carrying capacities of specimens WP20d-100 and WP20d-120 were lower than that of specimen W20d. Moreover, owing to the stable connection and high-strength grout layer between the wall panel and foundation beam inhibiting concrete damage around the wall toe, specimen WP35d-45 had excellent deformation and load-carrying capacities.

#### 4.2.2. Strain Measured in SBPDN Rebars (+25 mm)

**Figure 15:**
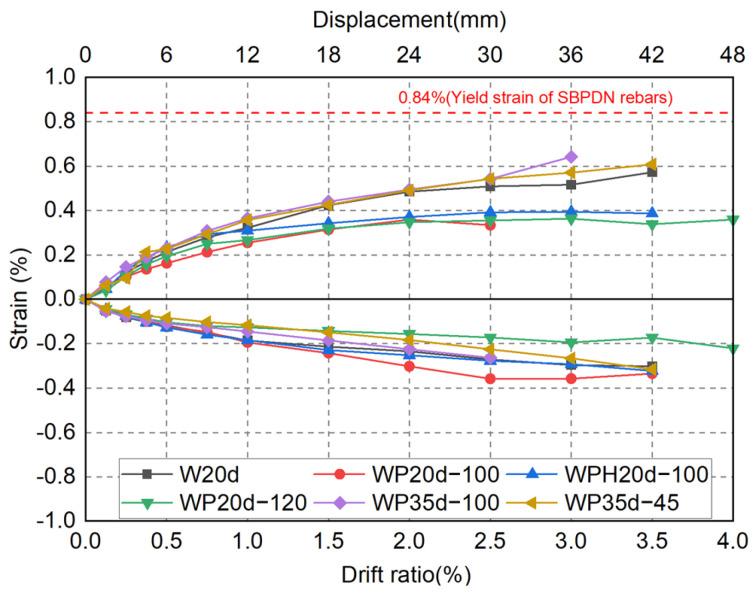
Envelopes of strain measured on SBPDN rebars.

The strains in tension and compression at a height of +25 mm of SBPDN rebars are summarized in Figure 15. The yield strain of the SBPDN rebars (0.84%) is indicated by a dashed red line. Since the compressive strength of concrete was much greater than its tensile strength, the concrete in the compression zone held part of the load without the influence of the sheath duct, resulting in the strain in the compression zone being smaller than that in the tension zone.

Similar to the hysteresis curves, the strain on the tensile side of specimens W20d, WP35d-100, and WP35d-45 displayed stable development as the drift ratio increased, which demonstrated that SBPDN rebars could provide the drift-hardening concrete walls with sufficient deformation capability until a large drift level. Conversely, since the sheath ducts were pulled out (1.5%), the strain of specimens WP20d-100, WPH20d-100, and WP20d-120 remained around 0.4% until the end of the tests.

### 4.3. Residual Drift Ratio

The residual drift ratio is an essential and comprehensive indicator recommended in the Federal Emergency Management Agency (FEMA) guidelines [34,35] to categorize damage states and specify the probability of repairability and restorability for earthquake-damaged building structures and/or components, including concrete structures, steel structures, and masonry structures. McCormick et al. [36] recommended applying the residual drift ratio to define limit stages in PBSD frameworks and particularly specified 0.5% as an acceptable residual drift ratio. Similarly, FEMA [35] proposed a 0.5% residual drift ratio as the critical limit, indicating a 98% repairable probability.

**Figure 16:**
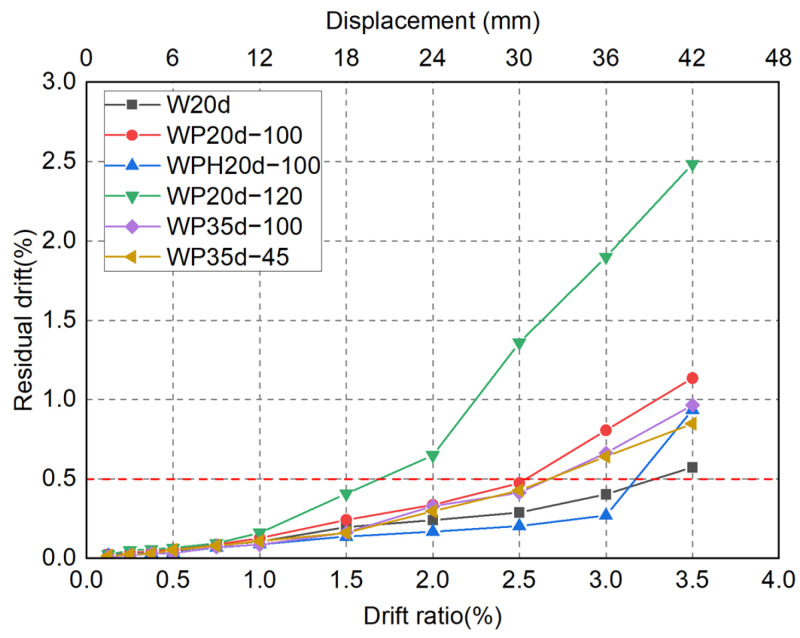
Residual deformation.

Figure 16 shows the average residual drift ratio of the initial push and pull directions measured at each targeted drift level. As obvious in Figure 16, the experimental residual drift ratios of all six specimens increased with the transient drift ratio until 1% and were kept below 0.5%. Owing to the instability of the connection, a sharp increase in residual drift ratio was observed in WP20d-120 beyond the drift ratio of 1.5%. As the damage to the wall toe concrete increased, the residual drift ratio of precast specimens WP20d-100, WP35d-100, and WP35d-45 became slightly greater than the CIP specimen W20d. As also seen in Figure 16, although WPH20d-100 exhibited a noticeable “rebounding” effect during the unloading stage of the test due to the higher axial load ratio, the instability of the connection mechanism was effectively suppressed compared to WP20d-100. As a result, its residual deformation remained slightly smaller than that of the CIP specimen, even up to the drift ratio of 3.0%.

### 4.4. Propagation of Crack Width

**Figure 17:**
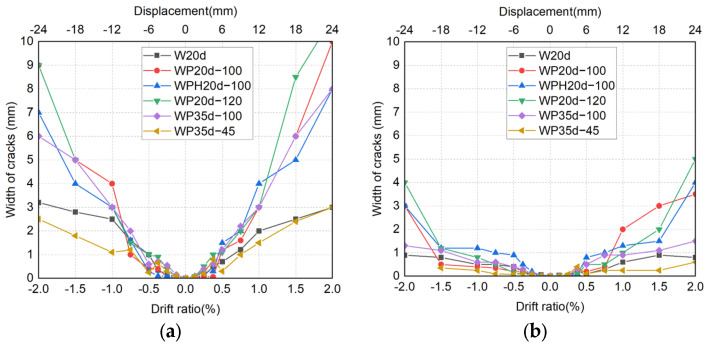
Propagation of crack: (**a**) loading; (**b**) unloading.

The crack width between the wall panel and the foundation beam is an important indicator used to evaluate the stability of the connection. Figure 17 shows the peak and residual widths located in the wall toe at each transient drift ratio of up to 2.0% for all specimens. The crack widths were measured using a crack width ruler whose range varies from 0.03 mm to 2.5 mm. It is noted that the measured crack widths at the drift ratio of 2.0% are only displayed as a reference because the crack widths measured at that drift ratio became sufficiently unreliable due to the influence of severe crushing and spalling off of the concrete.

As obvious in Figure 17a, based on whether the connections were stable, the cracks in the wall toe of six specimens developed in two different trends: for the CIP specimen W20d and precast specimen WP35d-45, both of the crack widths were controlled under 3 mm at a drift ratio of 2%. Conversely, the cracking width of the other four specimens increased rapidly from the early stage of the loading test. As can be seen in the four precast specimens, the trend of cracking decreased as the axial load increased but accelerated as the diameter of the sheath duct increased. Moreover, no significant correlation was found between the embedded length and crack development.

According to the comparison of residual crack widths in Figure 17b, it is particularly noteworthy that the residual widths of specimens W20d and WP35d-45 were less than 1.0 mm, even after unloading within the drift ratio of 2.0%, which is the upper limit at the minor damage state prescribed in the Japan Building Disaster Prevention Association (JBDPA) guideline [37] and the AIJ design guideline [4]. Due to the ribs of sheath ducts being stuck in concrete, the residual crack widths of specimens WP20d-100, WPH20d-100, and WP20d-120 were significantly larger than those of other specimens. For specimen WP35d-100, although no pullout phenomenon occurred within the drift ratio of 2%, it could be inferred that the surrounding concrete started to be damaged due to the insufficient bond strength and the width of the crack being larger than that of specimens W20d and WP35d-45 but smaller than those of WP20d-100, WPH20d-100, and WP20d-120.

### 4.5. Energy Dissipation

**Figure 18:**
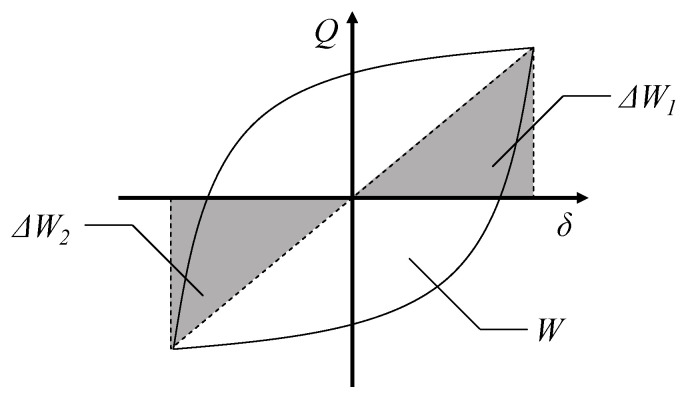
The definition of equivalent viscous damping coefficient *h_eq_*.

**Figure 19:**
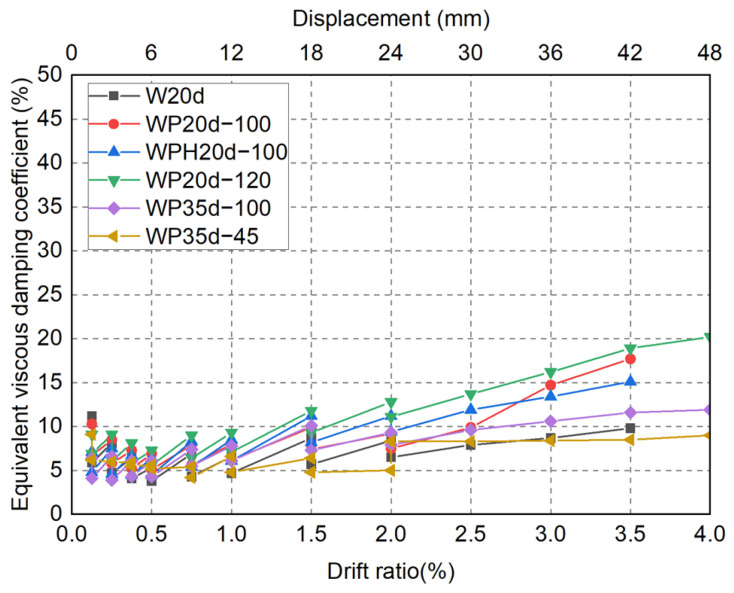
Measured equivalent viscous damping coefficients *h_eq_*.

To compare the energy-dissipation capacity of precast DHC walls, the equivalent viscous damping coefficient *h_eq_* proposed by Jacobsen [38,39] was used in this study. *h_eq_* is an indicator used to assess a component’s energy-absorption capacity based on the area of the hysteresis curve. A larger value indicates a stronger energy absorption capability, and the calculated method is shown in Figure 18 as well as Equations (1) and (2) as below:

(1) $$h_{eq}=\frac{1}{4\pi }\cdot \frac{W}{\left(\Delta W_{1}+\Delta W_{2}\right)/2}$$

(2) $$\Delta W=\frac{1}{2}Q\delta$$

where *Q* and *δ* are the lateral force and displacement of specimens, *W* is the energy consumption for a whole load cycle, and Δ*W*_1_ and Δ*W*_2_ are the stored energy in positive and negative parts of the load cycle. The measured equivalent viscous damping coefficients *h_eq_* are summarized in Figure 19.

As obvious in Figure 19, since there were two complete load cycles within the drift ratio of 2%, the first loading cycle with the same target drift ratio has a slightly higher energy-dissipation capacity than the second loading cycle due to the concomitant cracking of the concrete, giving the curve a jagged shape until the drift ratio of 2%.

Precast specimen WP35d-45 and CIP specimen W20d have similar levels of *h_eq_*, which demonstrated that the sheath ducts anchored in foundation beam stalely. The *h_eq_* of precast specimens WP20d-100, WPH20d-100, and WP20d-120 were 13%, 28%, and 36% higher than in the control group (W20d) since the sheath ducts were pulled out at the drift ratio of 1.5% and increased to 81%, 54%, and 93%, respectively, by the end of the loading test. Due to pullout not being obvious compared to other precast specimens, the *h_eq_* of specimen WP35d-100 was slightly higher than in the control group (about 10%).

Given the comparison mentioned above, it was demonstrated that increasing the diameter of the sheath duct could not enhance the stability of the connection but reduced the load-carrying capacity and repairability of building structures. On the other hand, anchoring SBPDN rebars separately could ensure the stability of connection until large deformation; however, the embedded length of 35d for SBPDN rebars may demand unnecessary depth for the adjacent beams, hindering the application of precast DHC walls to middle- and high-rise concrete buildings. Therefore, the optimal embedded length for sheath ducts needs to be clarified in a future study.

## 5. Bond Strength of Sheath Duct

To quantify the relationship between the surface of sheath duct and concrete, a formula for calculating the bond strength was applied in this study. Three basic assumptions were made in calculating the bond strength for the surface of sheath ducts: (1) no slippage between the grout and the inner surface of the sheath duct, (2) the stress on the SBPDN rebars could be transferred to the surface of the sheath ducts, and (3) the stress of SBPDN rebars could be calculated using the value of strain gauges and Hook’s law.

For the precast specimens for which the sheath ducts were pulled out, *τ*_*max*_ could be calculated using Equation (3), and the results are summarized in Table 3:

(3) $$\tau_{max}=\frac{{n\varepsilon_{max}E}_{s}A_{S}}{\pi D_{S}H_{S}}$$

In this equation, *n* is the number of SBPDN rebars, which equals four in this study, and *A*_s_ (=125 mm^2^) is the nominal area of each SBPDN rebar, and the corresponding Young’s modulus can be found in Table 2. *R_m_* is the drift ratio when the sheath ducts are pulled out. *ε*_*max*_ is the measured tensile strain of SBPDN rebars when the sheath duct became unstable. *A* is the side area of sheath ducts (*A* = *πD_s_H_s_*, *D*_s_, and *H*_s_ are the diameter and embedded length, respectively).

According to the calculated results shown in Table 4, the axial load ratio and embedded length have no effect on the bond strength of sheath ducts. The bond strengths of specimens WP20d-100, WPH20d-100, and WP35d-100 were stabilized at the same level and could be considered 4 N/mm^2^. On the other hand, in the calculated results of specimen WP20d-120, the bond strength was about 45% lower than the other three specimens. Since the heights of the ribs were the same for different sizes of sheath ducts, increasing the diameter of the sheath ducts decreases the ratio of the rib height to the diameter, which weakens the anchoring capacity of sheath ducts and thus reduces the bond strength of the surface.

## 6. Conclusions

In order to find a suitable size of sheath ducts and a reliable construction method for precast walls reinforced by SBPDN rebars, this study investigated the influence of the diameter, embedded length, and construction method of sheath ducts as well as the axial load ratio for precast DHC walls. A cast-in-place specimen and five precast specimens were fabricated and tested in combination with reversed lateral loading and constant axial load. The experimental observations, hysteresis behaviors, envelope curves, residual deformation, crack propagation, and energy dissipation are discussed in this paper. Moreover, in order to quantify the effect of the various experimental variables mentioned above, a formula was applied to calculate the bond strength of the sheath duct. Based on the experimental research introduced above, the following findings could be drawn:

1. Regardless of the construction method and axial load ratio, all the SBPDN rebars located in the boundary elements of the wall panel did not reach the yield strain, which demonstrates that SBPDN rebars could provide the DHC walls with sufficient drift-hardening capability until a large drift level (over 3%).
2. Compared to the CIP specimen, the precast specimens with shorter sheath ducts (20d) exhibited similar behavior until the drift ratio of 1.5%. From that drift on, the sheath duct started to become unstable, resulting in an increase in residual deformation and a decrease in lateral resistance.
3. Higher axial compression could enhance lateral resistance and suppress residual deformation; however, according to the calculated results in Section 5, axial compression had little influence on the bond strength of sheath ducts.
4. For the specimen with anchored SBPDN rebars separately in smaller sheath ducts (WP35d-45), it is noteworthy that both the load-carrying and deformation capacity are better than that of the cast-in-place specimen while exhibiting similar residual deformation, demonstrating the stability and effectiveness of the connection method. However, the embedded length of 35d for SBPDN rebars may demand an unnecessary depth for adjacent beams, and the suitable embedded length of sheath ducts needs to be clarified for practical engineering in a future study.
5. Based on the calculated bond strength of sheath ducts, increasing the embedded length is not a suitable solution to prevent sheath ducts from being pulled out. In addition, increasing the diameter of the sheath ducts decreases the ratio of the rib height to the diameter, which weakens the anchoring capacity of the sheath ducts and thus reduces the bond strength of the surface.

## Figures and Tables

**Table 1:** Test parameters and details of specimens.

Specimen	*f*’*_c_* (N/mm^2^)	*f*’*_g_* (N/mm^2^)	*ALRs*	Concentrated SBPDN Rebars		Longitudinal Rebars		Sheath Duct	
				Diameter	Length of Embedded	*ρ_wy_*	Type	Length of Embedded	Diameter (mm)
				(mm)	(mm)	(%)		(mm)	
W20d	36.0	-	0.075	U12.6-1	252 (20d)	0.70	D6-1	-	-
WP20d-100	36.1	56.8	0.075	U12.6-2	252 (20d)	0.70	D6-2	310	100
WPH20d-100	44.9	63.4	0.15	U12.6-2	252 (20d)	0.70	D6-2	310	100
WP20d-120	44.7	62.8	0.075	U12.6-2	252 (20d)	0.70	D6-2	310	120
WP35d-100	43.6	66.4	0.075	U12.6-2	441 (35d)	0.70	D6-2	510	100
WP35d-45	41.9	50.1	0.075	U12.6-3	441 (35d)	0.35	D6-3	460	45 × 4

*f*’*_c_* = concrete strength, *f*’*_g_* = grout strength, *ALRs* = axial load ratio; *p_w__y_* = the ratio of longitudinally distributed reinforcement (deformed rebars, D6).

**Table 2:** Mechanical properties of reinforcement.

Category	*E_s_*	*f_y_*	*ε_y_*	*f_u_*
	(kN/mm^2^)	(N/mm^2^)	(×0.01)	(N/mm^2^)
SD295A D6-1	192	402	0.21	524
SD295A D6-2	191	384	0.20	491
SD295A D6-3	192	388	0.22	489
SBPDN U12.6-1	217	1393 *	0.84 *	1467
SBPDN U12.6-2	212	1376 *	0.84 *	1459
SBPDN U12.6-3	217	1380 *	0.88 *	1461

*E_s_* = Young’s modulus, *f_y_* and *e_y_* = yield strength and corresponding strain, *f_u_* = tensile strength, * 0.2% offset yield strength and strain.

**Table 3:** Main experimental parameters of specimens.

Specimens	Initial Stiffness (*K*)	Peak Load (*V_max_*)	Peak Drift Ratio (*R_max_*)
	(kN/mm)	(kN)	rad (%)
W20d	50.7	−253	−3
WP20d-100	43.0	−233	−2.5
WPH20d-100	106.5	−248	−1.5
WP20d-120	56.8	−199	−1.5
WP35d-100	55.4	253	3
WP35d-45	55.2	296	3

**Table 4:** Bond strength on the surface of sheath ducts.

Specimen	*R_m_*	*ε_max_*	*A*	*τ_max_*
	rad(%)	(%)	(mm²)	(N/mm²)
WP20d-100	1.5	0.365	97,340	4.07
WPH20d-100	1.5	0.377	97,340	4.10
WP20d-120	1.5	0.310	116,808	2.81
WP35d-100	3.0	0.622	160,140	4.10

## Data Availability

The data are contained within the article.
